# Association Between Renal Sinus Fat and Cardiometabolic and Renin-Angiotensin System Parameters in Primary Aldosteronism

**DOI:** 10.1210/jendso/bvad154

**Published:** 2023-12-07

**Authors:** Ryunosuke Mitsuno, Kenji Kaneko, Toshifumi Nakamura, Daiki Kojima, Yosuke Mizutani, Tatsuhiko Azegami, Shintaro Yamaguchi, Yoshitake Yamada, Masahiro Jinzaki, Kenichiro Kinouchi, Jun Yoshino, Kaori Hayashi

**Affiliations:** Division of Endocrinology, Metabolism and Nephrology, Department of Internal Medicine, Keio University School of Medicine, Tokyo 160-8582, Japan; Division of Endocrinology, Metabolism and Nephrology, Department of Internal Medicine, Keio University School of Medicine, Tokyo 160-8582, Japan; Division of Endocrinology, Metabolism and Nephrology, Department of Internal Medicine, Keio University School of Medicine, Tokyo 160-8582, Japan; Division of Endocrinology, Metabolism and Nephrology, Department of Internal Medicine, Keio University School of Medicine, Tokyo 160-8582, Japan; Division of Endocrinology, Metabolism and Nephrology, Department of Internal Medicine, Keio University School of Medicine, Tokyo 160-8582, Japan; Division of Endocrinology, Metabolism and Nephrology, Department of Internal Medicine, Keio University School of Medicine, Tokyo 160-8582, Japan; Division of Endocrinology, Metabolism and Nephrology, Department of Internal Medicine, Keio University School of Medicine, Tokyo 160-8582, Japan; Department of Radiology, Keio University School of Medicine, Tokyo 160-8582, Japan; Department of Radiology, Keio University School of Medicine, Tokyo 160-8582, Japan; Division of Endocrinology, Metabolism and Nephrology, Department of Internal Medicine, Keio University School of Medicine, Tokyo 160-8582, Japan; Division of Endocrinology, Metabolism and Nephrology, Department of Internal Medicine, Keio University School of Medicine, Tokyo 160-8582, Japan; Division of Endocrinology, Metabolism and Nephrology, Department of Internal Medicine, Keio University School of Medicine, Tokyo 160-8582, Japan

**Keywords:** renal sinus fat, primary aldosteronism, renin-angiotensin system (RAS), hypertension, obesity, cardiometabolic function

## Abstract

**Context:**

Renal sinus fat (RSF) accumulation is associated with cardiometabolic diseases, such as obesity, diabetes, hypertension, and chronic kidney disease. However, clinical implications of RSF in primary aldosteronism (PA) remain unclear.

**Objective:**

We aimed to investigate relationships between RSF volume and key cardiometabolic and renin-angiotensin system (RAS) parameters in PA patients and clarify the differences in these relationships between unilateral and bilateral subtypes.

**Methods:**

We analyzed data obtained from well-characterized PA patients that involved 45 unilateral (median age: 52 years; 42.2% men) and 92 bilateral patients (51 years; 42.4% men).

**Results:**

RSF volume normalized by renal volume (RSF%) was greater in the unilateral group than in the bilateral group (*P* < .05). RSF% was greater in men than in women (*P* < .05). RSF% positively correlated with parameters related to cardiometabolic risk, including age, body mass index, visceral fat volume, creatinine, triglycerides/high-density lipoprotein cholesterol ratio, uric acid, fasting glucose, and C-reactive protein regardless of PA subtypes (all *P* < .05). Intriguingly, RSF% positively correlated with plasma aldosterone concentration (PAC), aldosterone-to-renin ratio, and intact parathyroid hormone (iPTH) (all *P* < .05) in bilateral patients but did not correlate with RAS parameters and even showed an opposite trend in unilateral patients. In subgroup analyses by sex, these distinctions became more evident in women. After adjustment for potential confounders, RSF% remained positively correlated with PAC and iPTH in bilateral patients.

**Conclusion:**

Our results indicate that RSF accumulation is involved in cardiometabolic dysfunction associated with PA. However, there were distinct correlations between RSF volume and RAS parameters according to sex and PA subtypes.

Primary aldosteronism (PA) caused by overproduction of aldosterone from the adrenal zona glomerulosa is now recognized to be the most common cause of secondary hypertension [1-3]. Data obtained from a recent meta-analysis demonstrate that PA prevalence ranges from 1.4% to 29.8% in hypertensive patients in the United States [4]. Aldosterone excess and subsequent activation of the mineralocorticoid receptor (MR) are involved not only in hypertension but also in obesity and its cardiometabolic complications, such as dyslipidemia, type 2 diabetes mellitus (T2DM), and chronic kidney disease (CKD) [5, 6]. In fact, PA patients have higher incidence of obesity-associated cardiometabolic diseases, such as metabolic syndrome, T2DM, dyslipidemia, CKD, and heart failure [7-9]. The mechanism explaining the relationships between PA and cardiometabolic abnormalities is unclear but likely involves altered distribution and function in adipose tissue. Indeed, recent studies suggest that visceral adipose tissue accumulation, a major risk factor of various cardiometabolic diseases [10, 11], is linked to cardiac dysfunction, renal dysfunction, and increased plasma aldosterone concentration (PAC) in PA patients [12-15]. In addition, data obtained from studies conducted in rodents and cultured cells demonstrate that adipocyte-derived factor(s) enhance aldosterone production in human adrenocortical cells and regulate endothelial function [16, 17] and that mature adipocytes isolated from human and mouse adipose tissue secret aldosterone, which, in turn, induces vascular dysfunction [18, 19]. Taken together, these findings highlight the importance of reciprocal interactions between aldosterone and adipocyte biology in the pathophysiology of PA-associated cardiometabolic complications.

Renal sinus fat (RSF) is an ectopic adipose tissue depot located at the medial border of the kidney where blood vessels, ureters, and lymphatic channels are embedded [20]. Data obtained from studies conducted in obese rodents suggest that RSF accumulation could deteriorate renal function and hemodynamics by physically compressing renal structures. Consistent with these experimental findings, recent cross-sectional studies have revealed the close relationships between increased RSF accumulation and cardiometabolic diseases, such as obesity [21], T2DM [22], hypertension [23, 24], and CKD [25, 26]. However, the relationships between RSF volume and cardiometabolic and renin-angiotensin system (RAS) parameters in PA patients remain unexplored.

The major purpose of the present study was to explore the clinical implications of RSF accumulation for the pathophysiology of PA. To this end, we retrospectively investigated the relationships between RSF volume measured by abdominal computed tomography (CT) and key cardiometabolic and RAS parameters in clinically well-characterized PA patients and clarified the differences in these relationships between unilateral and bilateral subtypes.

## Materials and Methods

### Study Participants

This single-center retrospective study, which was conducted in Keio University Hospital, Tokyo, Japan, was approved by the research ethics committee of Keio University School of Medicine (approval No. 20221032). We first assessed for the eligibility of 170 patients older than 20 years who underwent a confirmatory test (ie, captopril challenge test, furosemide-upright test, or saline infusion test) and diagnosed with PA, and those who underwent adrenal venous sampling (AVS) between July 1, 2018 and November 30, 2021. We next excluded patients with subclinical Cushing syndrome (n = 4) and those with Cushing syndrome (n = 2), and those who did not have a blood test for PAC at 7 Am (n = 17), and an abdominal to pelvic CT scan within 6 months (n = 9). We also excluded a patient who had a single kidney after surgery. As a result, this study included 137 patients.

### Clinical Data and Assay Methods

Body mass index (BMI) was calculated as body weight in kilograms divided by height in meters squared. PAC was measured using a radioimmunoassay (RIA; SPAC-S aldosterone kit, Fuji Rebio) from July 2018 to October 2018 or a chemiluminescent enzyme immunoassay (CLEIA) (Accuraseed aldosterone kit, FUJIFILM Wako Pure Chemical Corporation) from November 2018 to November 2021. Active renin concentration (ARC) was measured using an immunoradiometric assay (Renin IRMA-FR kit, Fuji Rebio) from July 2018 to October 2018 or a CLEIA (Accuraseed renin kit, FUJIFILM Wako Pure Chemical Corporation) from November 2018 to November 2021. Serum intact parathyroid hormone (1-84) (iPTH) concentration was determined using an enzyme immunoassay (EIA; E test TOSOH 2 intact PTH kit, Tosoh Bioscience) from July 2018 to November 2019 or a CLEIA (AIA-PACK CL intact PTH kit, Tosoh Bioscience) from December 2019 to November 2021. Characteristics of assays used in this study are provided in Supplementary Table S1 [27]. Estimated glomerular filtration rate (eGFR) was calculated using GFR estimation formula for the Japanese population [28].

### Diagnosis and Subtypes of Primary Aldosteronism

PA was diagnosed based on the guidelines of the Japan Endocrine Society [29] and the Japanese Society of Hypertension [30] as (1) a high aldosterone-to-renin ratio (ARR) (>40 with PAC expressed in pg/mL and ARC expressed in pg/mL) and (2) at least one positive result on a confirmatory test (ie, captopril challenge test, furosemide-upright test, or saline infusion test).

To determine whether aldosterone overproduction was lateralized in a unilateral adrenal gland, we performed AVS in the PA patients. Of the 137 patients who underwent AVS, 45 were diagnosed with unilateral PA and 92 were diagnosed with bilateral PA. The lateralized ratio was calculated by dividing the ratio of the PAC/serum cortisol concentration (PAC/F) in the ipsilateral adrenal vein by the ratio of PAC/F in the contralateral adrenal vein. The contralateral ratio was calculated by dividing the ratio of PAC/F in the contralateral adrenal vein by the ratio of PAC/F in the inferior vena cava. Lateralization of overproduction in the adrenal glands was indicated when the lateralized ratio was greater than or equal to 4 with adrenocorticotropin (ACTH) stimulation and when contralateral ratio was less than 1 with ACTH stimulation [31]. In cases where AVS was unsuccessful or difficult, the diagnosis was determined by a medical team that involved multiple experienced endocrinologists (T.N., Y.M., K.K.).

### Renal Sinus Fat Volume Measurement

All patients underwent multislice helical CT with a 1.2-mm slice thickness, starting from the upper edge of the liver to the pelvis. All imaging analyses were performed on a dedicated Advantage Workstation (version 3.2, GE Healthcare). Renal sinus fat (RSF) volume (cm^3^), visceral fat volume (VFV, cm^3^), and subcutaneous fat volume (SFV, cm^3^) were determined by a semiautomated method that required a slice-by-slice manual definition of borders on CT axial images, and each volume was obtained. The boundary of RSF was defined as a straight-line tracing across the opening of the renal sinus from the dimples in the two adjacent lobes based on visual inspection so that the surrounding abdominal adipose tissue would be excluded from this measurement [20]. The subcutaneous fat area was defined as adipose tissue between the skin and muscle. The visceral fat area was defined as intra-abdominal tissue with density in the fat attenuation range. The fatty tissue was defined between −200 and −40 Hounsfield units. RSF volume (cm^3^) to total renal volume (RV, cm^3^) were determined for each kidney. RSF% was calculated by dividing the sum of both RSF volume values by the sum of both RV values and used for statistical analyses.

### Statistical Analysis

Data are presented as median (interquartile range) for continuous variables and number and percentage for categorical variables. The comparisons between unilateral and bilateral groups were assessed in Mann-Whitney test for continuous variables and Fisher exact test for proportions. Spearman correlation coefficient (ρ) was calculated to evaluate correlations between RSF% and variables. Multivariable regression analysis was performed to examine correlations between log-transformed RSF% and the variables of interest. Data are presented as standardized coefficient (β) and 95% CI. All significance tests were 2-tailed, and *P* less than .05 was considered statistically significant. SPSS (version 28; IBM Corp) was used to perform all statistical analyses.

## Results

### Comparisons of Unilateral and Bilateral Primary Aldosteronism Patients

Table 1 summarizes the demographic and clinical characteristics of the 137 PA patients included in this study. Forty-five patients were diagnosed with unilateral PA (median age: 52 years; 42.2% men) and 92 patients were diagnosed with bilateral PA (51 years; 42.4% men). PAC, ARR, serum sodium concentration, prevalence of adrenal nodule, serum iPTH concentration, and urine albumin level were significantly higher, while ARC, and serum potassium concentration were lower in the unilateral PA group than in the bilateral PA group. There were no significant differences in other major cardiometabolic parameters between groups. The proportions of diabetes mellitus and cardiovascular disease, use of antihypertensive medication, and dose of calcium-channel blocker did not differ between groups (see Table 1 and Supplementary Table S2 [27]). None of the patients took an RAS inhibitor (angiotensin-converting enzyme inhibitor, angiotensin II receptor blocker, MR antagonist).

**Table 1.** Clinical characteristics in unilateral and bilateral primary aldosteronism patients

	Total	Unilateral	Bilateral	*P*
No.	137	45	92	
Age, y (n = 137)	51.0 (45.0-58.0)	52.0 (48.0-59.0)	51.0 (44.8-57.0)	.376
Men, n (%)	58 (42.3%)	19 (42.2%)	39 (42.4%)	≥.999
Height, cm (n = 137)	164.8 (159.6-169.6)	165.0 (157.2-168.9)	164.4 (159.9-170.5)	.320
Body weight, kg (n = 137)	67.6 (57.4-76.3)	67.2 (59.4-77.7)	68.9 (54.7-77.6)	.693
BMI, (n = 137)	24.4 (21.3-27.4)	24.8 (22.5-27.9)	24.5 (21.3-27.6)	.731
VFV, cm^3^ (n = 137)	2588 (1529-4084)	2970 (1837-4065)	2441 (1511-4288)	.475
SFV, cm^3^ (n = 137)	5767 (4342-7960)	5641 (4342-7940)	5768 (4405-7979)	.822
VFV/SFV ratio (n = 137)	0.41 (0.28-0.63)	0.43 (0.28-0.75)	0.40 (0.28-0.62)	.480
Diabetes mellitus, n (%)	7 (5.1%)	1 (2.2%)	6 (6.5%)	.426
Cardiovascular disease, n (%)	2 (1.5%)	1 (2.2%)	1 (1.1%)	.551
Antihypertensive medication, n (%)	123 (89.8%)	40 (89.9%)	83 (90.2%)	.773
Calcium-channel blockers	120 (87.6%)	40 (89.9%)	80 (87.0%)	>.999
α-Blockers	16 (11.7%)	8 (17.8%)	8 (8.7%)	.157
β-Blockers	3 (2.2%)	2 (4.4%)	1 (1.1%)	.251
RAS inhibitors	0 (0%)	0 (0%)	0 (0%)	>.999
SBP, mm Hg (n = 137)	146.0 (131.0-158.5)	146.0 (130.0-156.0)	144.0 (131.8-159.0)	.627
DBP, mm Hg (n = 137)	91 (82.0-97.5)	88.0 (79.0-96.0)	92.0 (83.0-97.3)	.250
ARC, pg/mL (n = 137)	2.00 (1.05-2.80)	1.80 (0.60-2.30)	2.05 (1.37-3.12)	.007
PAC, pg/mL (n = 137)	236.0 (175.0-330.0)	365.0 (268.0-495.0)	201.0 (156.8-250.5)	<.001
ARR (n = 137)	122.3 (72.7-223.1)	225.0 (138.5-811.7)	102.3 (60.6-150.5)	<.001
Adrenal nodule, n (%)	87 (63.5%)	42 (93.3%)	45 (48.9%)	<.001
Serum cortisol, μg/dL (n = 135)	14.3 (11.7-16.6)	15.3 (12.5-17.5)	14.0 (11.6-16.2)	.086
Plasma ACTH, pg/mL (n = 134)	25.9 (20.1-36.9)	29.9 (22.3-39.5)	25.3 (19.6-36.1)	.139
Serum iPTH, pg/mL (n = 137)	57.7 (47.7-80.6)	67.0 (52.5-101.5)	55.0 (46.1-73.0)	.002
Serum 1,25(OH)_2_ D_3_, pg/mL (n = 136)	70.0 (29.0-90.0)	72.0 (62.5-95.5)	68.0 (53.0-87.0)	.081
Plasma BNP, pg/mL (n = 136)	15.4 (9.3-26.3)	19.4 (11.7-27.2)	13.2 (8.5-23.7)	.089
Serum creatinine, mg/dL (n = 137)	0.74 (0.64-0.89)	0.77 (0.66-0.91)	0.73 (0.63-0.87)	.168
Serum BUN, mg/dL (n = 137)	13.4 (11.0-16.3)	12.1 (10.7-15.1)	13.8 (11.6-16.6)	.073
eGFR, mL/min/1.73 m^2^ (n = 137)	74.0 (66.5-83.0)	71.0 (63.0-78.0)	75.0 (68.8-83.3)	.070
Serum Na, mmol/L (n = 137)	141.3 (140.0-142.7)	142.3 (141.1-143.3)	141.0 (139.9-142.3)	.001
Serum K, mmol/L (n = 137)	3.70 (3.45-3.90)	3.40 (3.10-3.70)	3.80 (3.60-4.00)	<.001
Serum Ca, mg/dL (n = 137)	9.10 (8.80-9.30)	9.10 (8.75-9.40)	9.05 (8.83-9.30)	.502
Urine albumin, mg/g creatinine (n = 135)	9.4 (5.0-24.4)	15.3 (7.5-54.4)	7.30 (4.35-14.2)	<.001
Serum uric acid, mg/dL (n = 102)	5.15 (4.30-6.13)	5.10 (4.10-6.00)	5.20 (4.50-6.10)	.697
Serum total cholesterol, mg/dL (n = 102)	207.0 (187.0-226.8)	208.0 (189.0-225.3)	206.0 (187.8-226.0)	.877
Serum TGs, mg/dL (n = 89)	111.0 (75.0-165.0)	122.0 (92.5-160.8)	104.0 (69.5-169.0)	.280
Serum LDL cholesterol, mg/dL (n = 124)	116.0 (96.0-135.8)	118.0 (96.0-137.0)	115.5 (99.0-134.2)	.873
Serum HDL cholesterol, mg/dL (n = 125)	57.0 (47.0-68.5)	55.0 (46.0-66.5)	57.5 (46.1-71.5)	.567
TGs/HDL cholesterol ratio (n = 89)	1.95 (1.20-3.00)	2.29 (1.40-2.95)	1.79 (1.00-3.57)	.263
Fasting plasma glucose, mg/dL (n = 136)	101.0 (96.0-110.8)	101.0 (97.0-111.0)	100.0 (96.0-110.0)	.727
HbA_1c_, % (n = 136)	5.50 (5.30-5.70)	5.50 (5.30-5.80)	5.50 (5.30-5.70)	.816
Serum CRP, mg/dL (n = 110)	0.03 (0.02-0.06)	0.03 (0.02-0.10)	0.03 (0.01-0.06)	.490

Data are presented as median (interquartile range [IQR]) for continuous variables and number and percentage for categorical variables. The differences between unilateral and bilateral groups were assessed using Mann-Whitney test for continuous variables and Fisher exact test for proportions.

Abbreviations: 1,25(OH)_2_ D_3_; 1,25-dihydroxyvitamin D_3_, ACTH, adrenocorticotropin; ARC, active renin concentration; ARR, aldosterone-to-renin ratio; BMI, body mass index; BNP, brain natriuretic peptide; BUN, blood urea nitrogen; CRP, C-reactive protein; DBP, diastolic blood pressure; eGFR, estimated glomerular filtration rate; HbA_1c_, glycated hemoglobin A_1c_; HDL, high-density lipoprotein; iPTH, intact parathyroid hormone (1-84); LDL, low-density lipoprotein; PAC, plasma aldosterone concentration; RAS, renin-angiotensin system; SBP, systolic blood pressure; SFV, subcutaneous fat volume; TGs, triglycerides; VFV, visceral fat volume.

**Figure 1.**
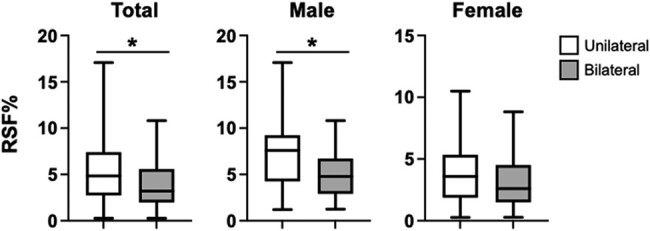
Comparison of RSF% between unilateral and bilateral primary aldosteronism (PA) patients. Renal sinus fat (RSF) volume normalized by renal volume (RSF%) was determined in the unilateral (n = 45) and bilateral (n = 92) PA patients for each sex. *P less than .05. *P* values were calculated using Mann-Whitney test.

Although BMI, VFV, SFV, or VFV/SFV ratio did not differ between groups, RSF% was significantly higher in the unilateral group than in the bilateral group (see Table 1 and Fig. 1). When we categorized patients according to sex, RSF% was significantly (*P* < .05) greater in men (5.36% [3.13%-7.50%]) than in women (3.02% [1.72%-4.86%]). In addition, male patients with unilateral PA had a significantly higher RSF% than those with bilateral PA, and female patients showed a similar trend (see Fig. 1).

### Associations Between Renal Sinus Fat Volume and Cardiometabolic and Renin-Angiotensin System Parameters in Unilateral and Bilateral Primary Aldosteronism Patients

**Figure 2.**
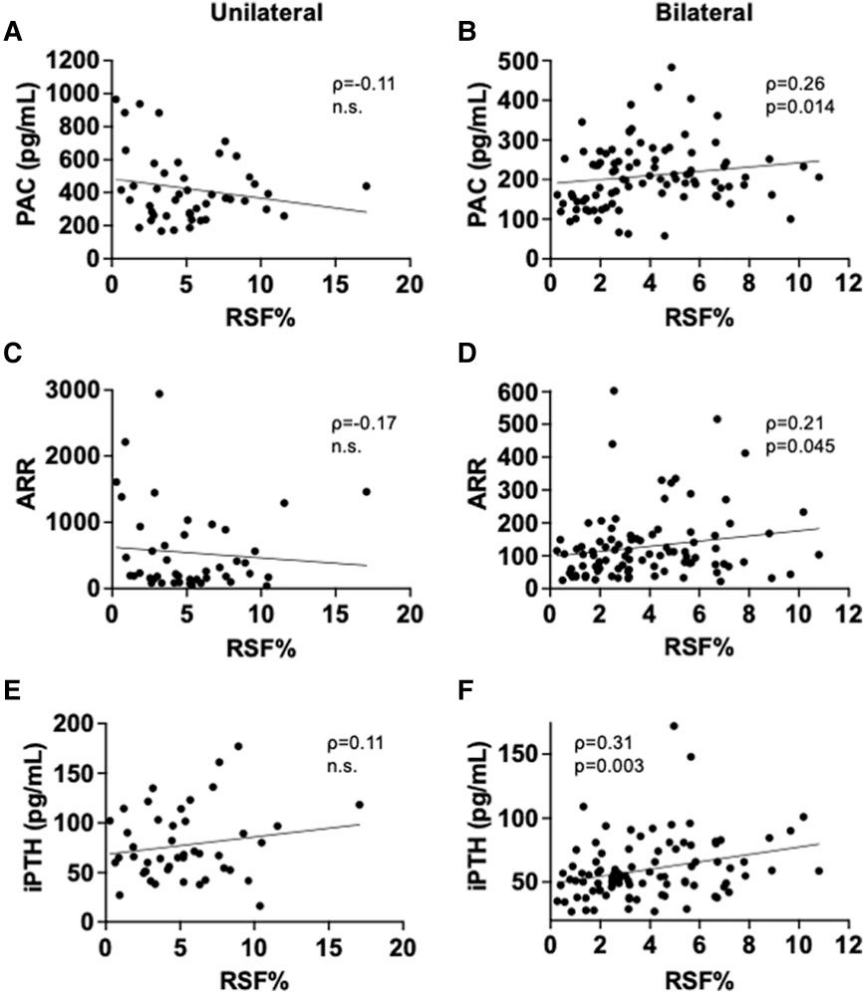
Associations between renal sinus fat volume normalized by renal volume (RSF%) and renal function and renin-angiotensin system (RAS) parameters in unilateral and bilateral primary aldosteronism (PA) patients. Relationships between RSF% and A and B, plasma aldosterone concentration (PAC); C and D, aldosterone-to-renin ratio (ARR); and E and F, serum intact parathyroid hormone (iPTH) concentration in unilateral (A, C, E) (n = 45) and bilateral (B, D, F) (n = 92) PA patients. Spearman correlation coefficient (ρ) and *P* value (*P*) are provided in each plot.

**Figure 3.**
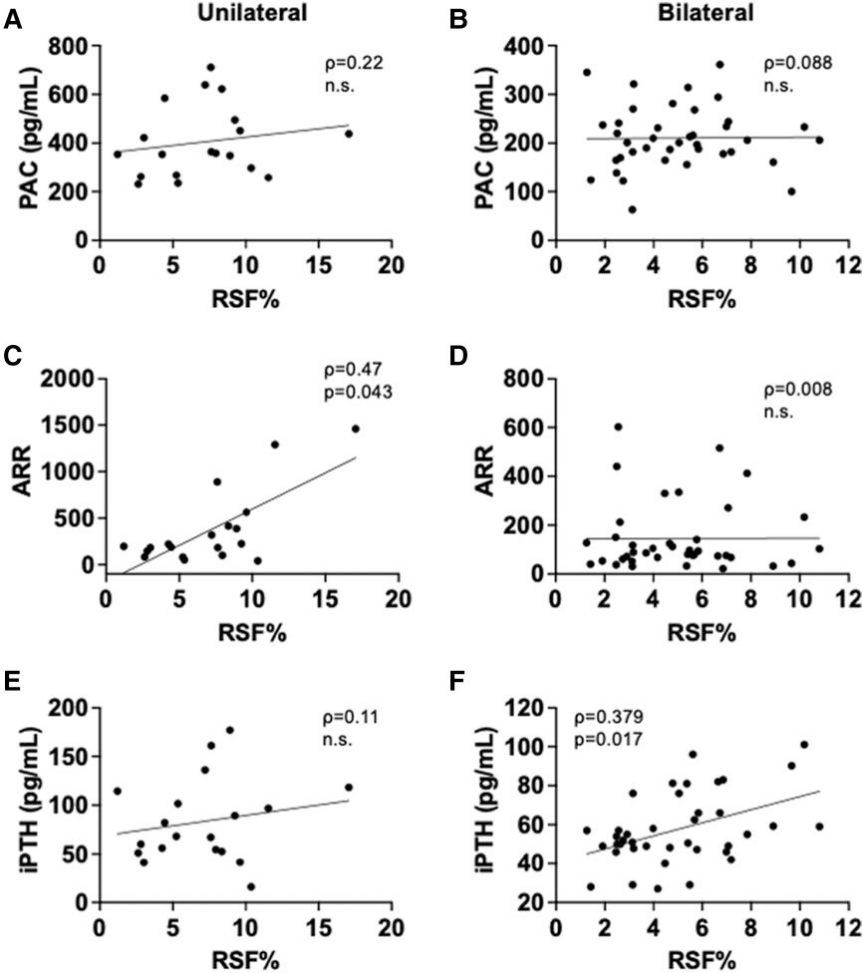
Associations between renal sinus fat volume normalized by renal volume (RSF%) and renal function and renin-angiotensin system (RAS) parameters in male patients. Relationships between RSF% and A and B, plasma aldosterone concentration (PAC); C and D, aldosterone-to-renin ratio (ARR); and E and F, serum intact parathyroid hormone (iPTH) concentration in unilateral (A, C, E) (n = 19) and bilateral (B, D, F) (n = 39) male patients. Spearman correlation coefficient (ρ) and *P* value (*P*) are provided in each plot.

**Figure 4.**
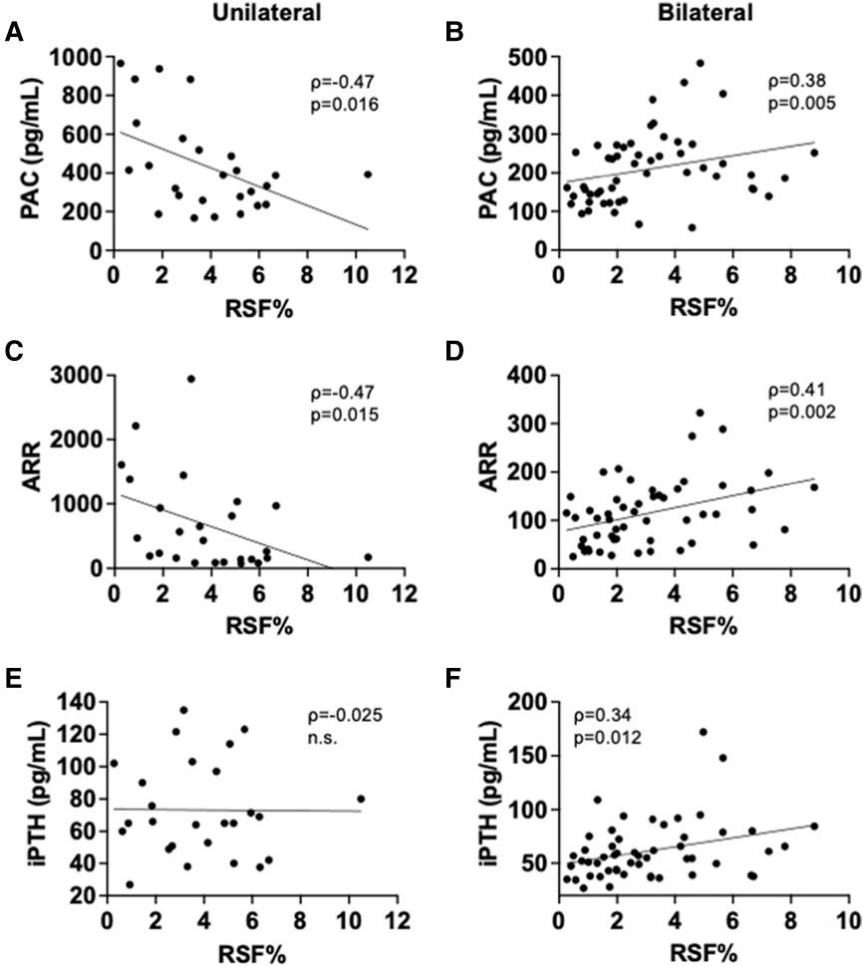
Associations between renal sinus fat volume normalized by renal volume (RSF%) and renal function and renin-angiotensin system (RAS) parameters in female patients. Relationships between RSF% and A and B, plasma aldosterone concentration (PAC); C and D, aldosterone-to-renin ratio (ARR); and E and F, serum intact parathyroid hormone (iPTH) concentration in unilateral (A, C, E) (n = 26) and bilateral (B, D, F) (n = 53) female patients. Spearman correlation coefficient (ρ) and *P* value (*P*) are provided in each plot.

We next evaluated the associations between RSF volume and cardiometabolic and RAS parameters in PA patients (Table 2). RSF% was positively associated with various parameters related to cardiometabolic risk, such as age, BMI, VFV, VFV/SFV ratio, serum creatinine concentration, serum uric acid concentration, triglycerides (TGs)/high-density lipoprotein (HDL) cholesterol ratio [32], fasting glucose concentration, and serum C-reactive protein (CRP) concentration, while it was negatively associated with serum HDL cholesterol concentration in PA patients regardless of its subtype (see Table 2). RSF% was also significantly correlated with serum TG concentration, eGFR, and glycated hemoglobin A_1c_ (HbA_1c_) in PA patients although there were some discrepancies between subtypes. The relationships between RSF% and these cardiometabolic parameters were overall more robust in women than in men (Supplementary Table S3 [27]). Intriguingly, RSF% positively correlated with PAC, and serum concentration of iPTH, which is a regulator of aldosterone production and cardiovascular function [33, 34] (see Table 2). However, there were distinct correlations between RSF% and key RAS parameters according to PA subtypes and sex. Specifically, RSF% was positively correlated with PAC, ARR, and serum iPTH concentration in bilateral patients whereas it did not correlate with RAS parameters and even showed an opposite trend in the unilateral patients (see Table 2 and Fig. 2). In the subgroup analyses by sex, we found positive relationships between RSF% and PAC and ARR in bilateral women but not in men (Figs. 3 and 4 and Supplementary Tables S3 and S4 [27]). There was a positive correlation between RSF% and serum iPTH concentration both in bilateral male and female patients. In contrast, although RSF% was positively correlated with ARR in unilateral men, it was negatively correlated with PAC and ARR in unilateral women (see Figs. 3 and 4 and Supplementary Tables S3 and S4 [27]). We further explored the associations between RSF volume and key RAS parameters using multivariable linear regression models. After adjusting for potential confounders such as age, sex, antihypertensive medication use, and BMI or VFV, RSF% remained positively correlated with PAC and serum iPTH concentration in bilateral patients (Table 3). In the subgroup analyses by sex, after inclusion of potential confounders, RSF% remained positively correlated with PAC, ARR, and serum iPTH concentration in bilateral women and it was negatively correlated with PAC and ARR in unilateral women (Supplementary Table S5 [27]).

**Table 2.** Associations between renal sinus fat volume normalized by renal volume and cardiometabolic and renin-angiotensin system parameters in unilateral and bilateral primary aldosteronism patients

	Total		Unilateral		Bilateral	
	ρ	*P*	ρ	*P*	ρ	*P*
Age, y (n = 137)	0.34	<.001	0.32	.033	0.35	<.001
BMI, (n = 137)	0.45	<.001	0.46	.002	0.45	<.001
VFV, cm^3^ (n = 137)	0.65	<.001	0.69	<.001	0.61	<.001
SFV, cm^3^ (n = 137)	0.36	<.001	0.31	.041	0.38	<.001
VFV/SFV ratio (n = 137)	0.56	<.001	0.69	<.001	0.47	<.001
SBP, mm Hg (n = 137)	0.15	.074	0.25	.103	0.13	.217
DBP, mm Hg (n = 137)	0.11	.209	0.26	.090	0.06	.564
ARC, pg/mL (n = 137)	−0.03	.690	−0.18	.246	−0.11	.284
PAC, pg/mL (n = 137)	0.22	.009	−0.11	.469	0.26	.014
ARR (n = 137)	0.15	.088	−0.17	.254	0.21	.045
Serum cortisol, μg/dL (n = 135)	−0.04	.621	0.04	.780	−0.12	.265
Plasma ACTH, pg/mL (n = 134)	0.03	.703	0.03	.835	0.00	.973
Serum iPTH, pg/mL (n = 137)	0.26	.003	0.11	.466	0.31	.003
Serum 1,25(OH)_2_ D_3_, pg/mL (n = 136)	−0.38	.662	0.07	.637	−0.12	.268
Plasma BNP, pg/mL (n = 136)	0.00	.990	−0.07	.669	0.01	.937
Serum creatinine, mg/dL (n = 137)	0.38	<.001	0.46	.002	0.30	.004
Serum BUN, mg/dL (n = 137)	0.07	.421	−0.03	.855	0.14	.181
eGFR, mL/min/1.73 m^2^ (n = 137)	−0.23	.006	−0.22	.156	−0.20	.060
Serum Na, mmol/L (n = 137)	0.21	.014	0.04	.810	0.23	.027
Serum K, mmol/L (n = 137)	−0.08	.340	0.00	.980	0.01	.940
Serum Ca, mg/dL (n = 137)	−0.06	.519	−0.03	.844	−0.12	.243
Urine albumin, mg/g creatinine (n = 135)	0.09	.310	0.12	.425	0.01	.898
Serum uric acid, mg/dL (n = 102)	0.46	<.001	0.61	<.001	0.39	.001
Serum total cholesterol, mg/dL (n = 102)	0.05	.616	0.15	.429	−0.01	.963
Serum TGs, mg/dL (n = 89)	0.31	.003	0.41	.024	0.23	.083
Serum LDL cholesterol, mg/dL (n = 124)	0.06	.504	0.10	.538	0.01	.907
Serum HDL cholesterol, mg/dL (n = 125)	−0.38	<.001	−0.36	.023	−0.36	<.001
TGs/HDL cholesterol ratio (n = 89)	0.36	<.001	0.45	.013	0.27	.035
Fasting plasma glucose, mg/dL (n = 136)	0.29	<.001	0.45	.002	0.22	.033
HbA_1c_, % (n = 136)	0.18	.032	0.05	.733	0.24	.020
Serum CRP, mg/dL (n = 110)	0.35	<.001	0.39	.009	0.29	.020

Spearman correlation coefficient (ρ) was calculated to evaluate correlations between renal sinus fat volume normalized by renal volume and cardiometabolic and RAS parameters in unilateral (n = 45) and bilateral (n = 92) primary aldosteronism patients.

Abbreviations: 1,25(OH)_2_ D_3_; 1,25-dihydroxyvitamin D_3_, ACTH, adrenocorticotropin; ARC, active renin concentration; ARR, aldosterone-to-renin ratio; BMI, body mass index; BNP, brain natriuretic peptide; BUN, blood urea nitrogen; CRP, C-reactive protein; DBP, diastolic blood pressure; eGFR, estimated glomerular filtration rate; HbA_1c_, glycated hemoglobin A_1c_; HDL, high-density lipoprotein; iPTH, intact parathyroid hormone (1-84); LDL, low-density lipoprotein; PAC, plasma aldosterone concentration; RAS, renin-angiotensin system; SBP, systolic blood pressure; SFV, subcutaneous fat volume; TGs, triglycerides; VFV, visceral fat volume.

**Table 3.** Multivariable regression models exploring the associations between log-transformed renal sinus fat volume normalized by renal volume and renin-angiotensin system parameters in bilateral primary aldosteronism patients

	Model 1		Model 2		Model 3	
	β (95% CI)	*P*	β (95% CI)	*P*	β (95% CI)	*P*
PAC, pg/mL	0.24 (0.12 to 0.47)	.008	0.25 (0.13 to 0.50)	<.001	0.27 (0.09 to 0.36)	<.001
ARR	0.13 (–.05 to 0.26)	.422	0.08 (–0.08 to 0.17)	.301	0.10 (–0.04 to 0.20)	.195
Serum iPTH, pg/mL	0.34 (0.17 to 0.50)	<.001	0.24 (0.09 to 0.39)	.004	0.21 (0.03 to 0.34)	.013

Model 1 adjusted for age and sex. Model 2 adjusted for age, sex, antihypertensive medication use, and BMI. Model 3 adjusted for age, sex, antihypertensive medication use, and VFV.

Abbreviations: β, adjusted β coefficient; ARR, aldosterone-to-renin ratio; BMI, body mass index; iPTH, intact parathyroid hormone; PAC, plasma aldosterone concentration; VFV, visceral fat volume.

## Discussion

In present study, we explored the clinical implications of RSF accumulation in the pathophysiology of PA and analyzed data obtained from clinically well-characterized PA patients. We identified robust correlations between RSF accumulation and various parameters related to cardiometabolic risk, including age, BMI, VFV, VFV/SFV ratio, TGs/HDL-C [32], uric acid, fasting glucose, and CRP in PA patients regardless of subtype. In addition, our findings that RSF% is associated with increased serum creatinine concentration and decreased eGFR in PA patients are highly consistent with data from previous studies that have demonstrated the relationship between RSF volume and renal dysfunction in patients with obesity and T2DM [35], hypertension [26], and CKD [25, 26]. Taken together, these findings suggest that RSF accumulation is involved in the mechanisms explaining higher incidence of cardiometabolic diseases, such as metabolic syndrome, T2DM, dyslipidemia, CKD, and cardiovascular disease [5, 6], although we do not refute the possibility that cardiometabolic dysfunction could affect RSF development in PA patients.

RSF accumulation was positively correlated with PAC in the bilateral PA patients even after accounting for key confounders such as age, sex, antihypertensive medication use, and BMI or VFV. Due to the observational nature of the retrospective study, we are unable to determine whether the relationships are causal. However, these results may support the hypothesis that RSF-derived factor(s) such as leptin could bilaterally stimulate aldosterone production from the adrenal cortex anatomically located close to the renal sinus [16, 17]. An alternate, but not mutually exclusive, hypothesis is that RSF accumulation physically blocks the intrarenal outflow and elevates renal interstitial hydrostatic pressure [36, 37], which could contribute to the development of local and systemic RAS activation. Indeed, it was recently reported that RSF% tends to be correlated with an increase in urine angiotensinogen creatinine ratio in CKD patients [25]. In addition, RSF% was highly correlated with serum iPTH concentration, another RAS-associated parameter involved in cardiometabolic dysfunction [33, 34] in the bilateral PA patients. We hypothesize that RSF-derived leptin could contribute to increased PTH secretion from the parathyroid gland [38]. Given that aldosterone regulates PTH secretion through angiotensin II receptor 1 and MR [34], RSF-mediated local and systemic RAS activation also could increase PTH production. Indeed, PA patients have elevated concentrations of PTH and increased incidence of — and more severe — abdominal aortic calcification [39, 40]. In addition, higher PAC exacerbates the effect of PTH on cardiovascular death and vice versa [34]. Taken together, our results indicate RSF accumulation could be involved in cardiovascular dysfunction induced by higher PAC and PTH.

In contrast, RSF volume did not correlate with RAS parameters and even showed an opposite trend in the unilateral patients. We also found significant inverse relationships between RSF% and PAC and ARR in the unilateral women even after adjustment for key confounders. Our findings are consistent with previous studies that found epicardial fat thickness and visceral adipose tissue area are negatively associated with PAC and ARR in PA patients with unilateral aldosterone-producing adenoma [15, 41]. Although the mechanism responsible for our observation of the dissociation between unilateral and bilateral groups remains unclear, it is possible that abnormally high PAC causes adipose tissue inflammation and fibrosis, leading to the inhibition of RSF expansion in unilateral PA patients [42]. Data obtained from studies conducted in rodents show that aldosterone causes inflammation and fibrotic changes in various organs including adipose tissue [43, 44]. Indeed, it was recently reported that aldosterone-producing adenoma patients have higher expression of proinflammatory cytokines and fibrosis markers, such as interleukin-6, tumor necrosis factor-α, fibronectin, and transforming growth factor-β-1, in perirenal and subcutaneous adipose tissue depots than normotensive people and patients with essential hypertension [45].

We found RSF% was greater in PA male than in PA female patients, which is consistent with data obtained from the recent studies conducted in non-PA populations [21, 25, 46]. In addition, relationships between RSF% and cardiometabolic and RAS parameters were overall stronger in women than in men regardless of subtypes. The mechanisms explaining these sexual dimorphisms remain unclear but could involve sex hormones including testosterone and estrogen, both of which are known to directly regulate adipogenesis in rodents and cultured cells [47, 48]. It is therefore possible that sex hormones may affect RSF development independently of PAC and thus confound the relationships between RSF accumulation and RAS parameters in PA patients. Future large-scale studies are needed to further investigate the independent and combined effects of PA subtypes and sex on these relationships.

There are several limitations in our study. First, this study was conducted at a single center with a limited sample size, which could reduce the generalizability of the findings and the statistical power. Second, we examined only PA patients and it still remains unclear whether RSF volume is associated with RAS parameters in other patient populations. Third, we did not evaluate cardiometabolic parameters that could confound the relationships between RSF volume and RAS parameters, such as cardiac structure and function, endothelial function, and plasma leptin concentration [16]. Finally, the effect of an MR antagonist on RSF volume remains unexplored. Data obtained from studies conducted in cultured cells demonstrate that MR antagonists inhibits adipocyte differentiation [49, 50]. Therefore, it would be great of importance to determine whether an MR antagonist decreases RSF volume and whether alteration in RSF volume is associated with cardiometabolic benefits induced by an MR antagonist.

In conclusion, the results from the present study demonstrate that RSF volume is associated with key parameters related to cardiometabolic risk in PA patients regardless of its subtype. However, there are distinct correlations between RSF% and key RAS parameters, namely PAC, ARR, and serum iPTH concentration according to sex and PA subtypes. Future studies that involve genetically engineered animal models are awaited to determine the causal relationship between RSF accumulation and cardiometabolic abnormalities and further elucidate the complex, reciprocal relationships among adipocyte biology, RAS, and cardiometabolic function.

## Data Availability

The data underlying this article will be shared on reasonable request to the corresponding author.

## Abbreviations

1,25(OH)_2_ D_3_: 1,25-dihydroxyvitamin D_3_
ACTH: adrenocorticotropin
ARC: active renin concentration
ARR: aldosterone-to-renin ratio
AVS: adrenal venous sampling
BMI: body mass index
BNP: brain natriuretic peptide
BUN: blood urea nitrogen
CKD: chronic kidney disease
CLEIA: chemiluminescent enzyme immunoassay
CRP: C-reactive protein
CT: computed tomography
DBP: diastolic blood pressure
eGFR: estimated glomerular filtration rate
HbA_1c_: glycated hemoglobin A_1c_
HDL: high-density lipoprotein
iPTH: intact parathyroid hormone (1-84)
LDL: low-density lipoprotein
MR: mineralocorticoid receptor
PA: primary aldosteronism
PAC: plasma aldosterone concentration
RAS: renin-angiotensin system
SBP: systolic blood pressure
SFV: subcutaneous fat volume
T2DM: type 2 diabetes mellitus
TGs: triglycerides
VFV: visceral fat volume
